# A Numerical Investigation into the Effects of Overweight and Obesity on Total Knee Arthroplasty

**DOI:** 10.1155/2017/1496379

**Published:** 2017-05-29

**Authors:** Changjiang Wang, Yuan Guo, Junfen Shi, Weiyi Chen

**Affiliations:** ^1^Institute of Applied Mechanics and Biomedical Engineering, Taiyuan University of Technology, Taiyuan, Shanxi 030024, China; ^2^School of Engineering and Informatics, University of Sussex, Brighton BN1 9QJ, UK; ^3^Bioengineering Science Research Group, University of Southampton, Southampton SO17 1BJ, UK

## Abstract

Overweight and obesity increase risks of knee osteoarthritis, which is a major cause of disability. Severe knee osteoarthritis can be treated by knee arthroplasty. Total knee arthroplasty has been used in overweight and obese patients; however, clinical reports showed that the outcome of this group of patients was not good as normal-weight patients. Two computer models were created in this paper to simulate the effect of excess loads on the distal femoral bone and contact pressures in total knee arthroplasty during a gait cycle. The numerical results showed increased stress in periprosthetic distal femoral bones and higher contact pressure on tibial polyethylene insert during the stance phase. Based on the computer simulation results and published research work, cementless total knee arthroplasty with thicker tibial polyethylene insert may be a better option for overweight patients.

## 1. Introduction

Obesity has reached epidemic proportions globally, with more than 1.9 billion adults were overweight in 2014 according to World Health Organization and at least 600 million of them are clinically obese. Overweight is a body mass index (BMI) greater than or equal to 25 kg/m^2^, obesity is a BMI greater than or equal to 30 kg/m^2^, and morbid obesity is a BMI greater than or equal to 40 kg/m^2^. Obesity significantly increases the risk of developing numerous medical conditions including osteoarthritis, which is the commonest cause of severe disability among older people in the UK and North America [1]. Longitudinal data have shown that obesity is a powerful risk factor for the development of knee osteoarthritis; for every 5 kg increase in weight, the risk increases by 30% [2]. Leung et al. [3] studied the association between body mass index and risk of total knee replacement; they concluded that BMI is one of the most important predictors of risk of knee osteoarthritis and the risk of total knee replacement.

Osteoarthritis of the knee can be treated either conservatively or surgically. Currently, the main surgical intervention is knee replacement, and whilst highly effective for most patients, all joint replacements will fail in time requiring revision [1]. Studies to date report conflicting results related to the impact of obesity on total knee arthroplasty (TKA) outcomes. A number of studies reported that obesity negatively impacts on outcomes following TKA, specifically significantly increasing the need for revision surgery [4], increasing cost of rehabilitation [5], and reducing the survivorship of the prosthesis [6] and focal osteolysis [7]. Conversely, other studies reported that outcomes between obese and nonobese patients following TKA are comparable, specifically in terms of rate of complications and knee function [8] and general and disease-specific measures of health and function [9]. Foran et al. [10] reported that the change of Knee Society Score (KSS) which assesses knee pain and function in obese patients after total knee replacement is about 20% less than that of nonobese patients. Deakin et al. [11] in 2017 reported that when compared to obese and nonobese patients, morbidly obese patients undergoing TKA had a four points lower Oxford knee score.

A common public misconception is that osteoarthritis leads to obesity and that the surgical treatment (joint replacement) of this disabling joint disease leads to patient weight loss. Booth [12] reported that only 18% of obese patients lose weight after joint replacement. Recent study by Schwartsmann et al. [13] from postoperative analysis showed that patients undergoing TKA only reduce the BMI slightly, 0.08 kg/m^2^. Therefore, it is important to study the effect of excess weight on the total knee replacement after the operation.

Gait analysis on obese individuals has identified kinematic adaptations including slower velocity, shorter step length, increased double support time, decreased knee range of motion, and larger ground reaction forces in the obese compared to lean individuals [14]. Spyropoulos et al. [15] compared the movement of the hip, knee, and ankle in obese and normal-weight men while the subjects walked over level ground at their preferred speed. Obese adults were found to adopt a slower walking velocity than nonobese subjects during testing. DeVita and Hortobagyi [16] tested the effects of obesity on lower extremity joint kinetics and energetics during walking by analysing the motion of obese healthy adults and lean adults. They found obese participants used altered gait biomechanics and had less knee torque and power at their self-selected walking speed and equal knee torque and power while walking at the same speed as lean individuals.

Physiological loads giving rise to implant-bone relative micromovements of the order of 100 or 200 *μ*m may inhibit bone in-growth, resulting in the formation of a fibrous tissue layer around the prosthesis and eventually promoting loosening of the implant [17]. Taylor and Tanner [18] proposed that migration of implant is due to the progressive failure of the supporting cancellous bone. The degree of implant migration is dependent on the initial mechanical environment and can be determined using patient-specific finite element analysis [19].

Au et al. [20] developed a three-dimensional finite element model to study bone and interface stresses for four different tibial prosthesis designs. All implant models demonstrated a reduction of cancellous bone stress plus high compression beneath the central fixation posts. Shi et al. [21] developed a finite element model and studied the contact pressure on two types of total knee replacement; they identified the effects of malalignment and different loadings on the outcome of total knee replacement.

Walker et al. [22] measured the quantitative changes in bone mineral density (BMD) in the distal femur after cemented total knee arthroplasty in osteoarthritis knee joints. An average decrease in bone density of 17.1% was measured adjacent to the prosthesis at the 12-month follow-up examination. Bone loss was most rapid during the first 3 months after TKA. Spittlehouse et al. [23] reported the greatest BMD decrease of 16% in the distal anterior femur over the first 6-month postoperative period in 16 patients with uncemented knee prostheses.

The exercise rehabilitation regimen adopted immediately after a TKA is a very important factor because of the known relationship between osseointegration and implant micromotion. Booth [12] suggested that modified postoperative regimens were needed for obese patients, but there was a lack of research indicating the modification required in the exercise regimen. Bizzini et al. [24] reported, using a random sample, that supplementing an exercise rehabilitation regimen with an adapted agility and perturbation training programme can allow patients to regain better functions in the activities of daily living within the first 3 postoperative months. However, there is still a scarcity of research relating to obese and morbidly obese patients.

The most common reasons for TKR revisions are polyethylene wear and aseptic loosening. The generation of polyethylene wear particles and the resulting osteolysis is a cause of long-term loosening of TKR joint. Because of the change of stress status postoperatively, bone remodelling after TKA is also an important factor causing malalignment and loosening. Therefore, the effect of overweight and obesity on the TKA needs to be studied; in the paper, TKA components were modelled within a lower limb during a gait cycle, and the effect of excess weight on the stresses in the distal femoral periprosthetic bone were investigated. The changes of contact pressures on tibial polyethylene insert due to excess weight were also simulated.

## 2. Methods

### 2.1. Computer Models

**Figure 1:**
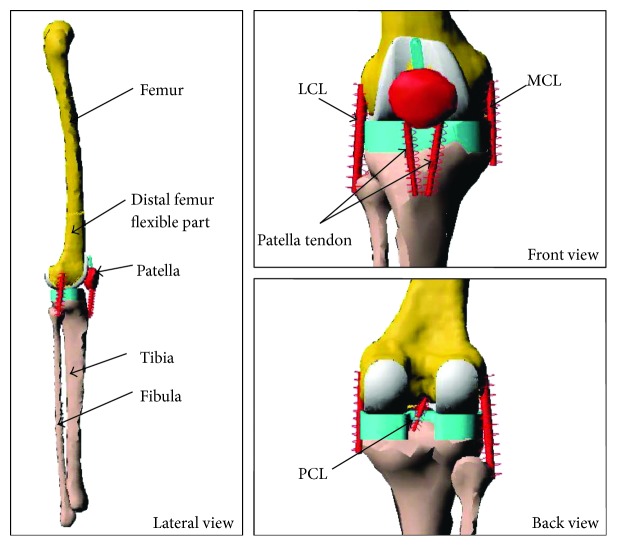
Finite element model of a total knee arthroplasty in a lower limb.

**Figure 2:**
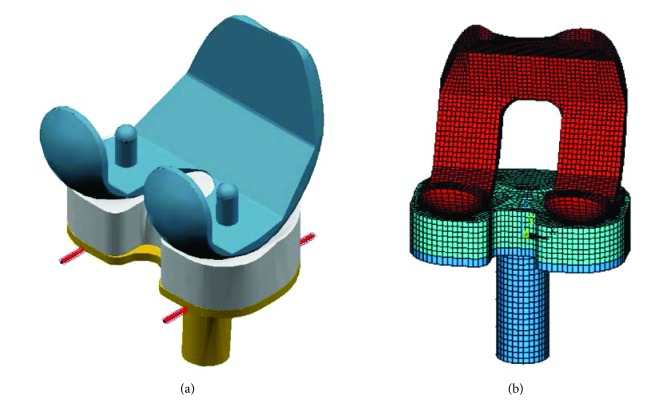
Finite element models of total knee arthroplasty components: (a) TKA computer model and (b) finite element mesh of the model.

Two computer dynamic models were created using MSC/ADAMS and used to study the effect of overweight on the distal femur and implants. Computer model one (CM1) shown in Figure 1 was used to investigate the influence of body weight on stress distribution in the distal femoral bone during a gait cycle. The geometrical models of the lower limb in this computer simulation model were obtained from the University of Brussels website [25]. The TKR implant used in the simulation is based on PFC Sigma system implant. The lower limb bone models in STL format were imported into MSC/ADAMS. The CM1 consists of anatomically correct bone models of the femur, tibia, fibula, and patella. The ligaments were added in anatomically correct location, and the knee prosthesis were inserted to ensure satisfactory alignment. Zero degree posterior slope of tibial tray was created to assemble the implant. The computer model two (CM2) shown in Figure 2 was used to study implant contact pressures related to wear testing and investigate the influence of overweight on the contact pressure in the tibial bearing component. The CM2 has six degrees of freedom, three translations, and three rotations of the knee joint. The femoral component was allowed to move vertically in the inferior-superior direction to rotate about a frontal axis to simulate valgus and varus rotation and to rotate about a transverse axis to simulate flexion and extension. The tibial components were allowed to translate in the anterior-posterior (AP) and medial-lateral (ML) directions and rotate about a fixed vertical axis located in the middle of the tibial condyles to simulate internal-external (IE) rotation. In CM2, the total AP translation restraint spring stiffness is 30 N/mm. The coefficient of friction between the femoral component and tibial bearing component is 0.04.

### 2.2. Loadings and Material Properties

**Figure 3:**
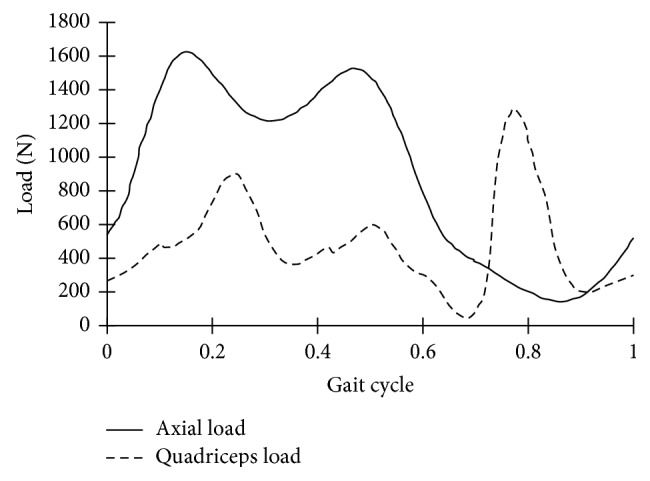
Hip axial (vertical) and quadriceps load during a gait cycle.

The first loading case is shown in Figure 3 and referred as normal weight loading; hip vertical and quadriceps load during a gait cycle were applied on CM1. The load data was adopted from published work [26–28]. A quadriceps force balances the vertical load through the patella ligament. The loading cases of 1.5 and 2 times normal weight were simulated by applying 1.5 and 2 times vertical load and quadriceps load, respectively. Stress distributions in the distal femur under different body weights can be obtained and compared. Boundary conditions on CM1 were applied to reproduce the Purdue knee simulator environments [27]. The Purdue simulator applies a vertical load and a flexion angle at a simulated hip and controls the horizontal AP and ML ankle translation. Rotation of the ankle in all directions is allowed in the computer model. The inputs to this computer model include vertical axial load on hip, quadriceps force, tibio-femoral AP translation, and AP and ML ankle translation. Loads and material properties used in this computer model are the same as those used in the published paper [21].

With CM2, a gait cycle was simulated; the time histories for the axial force, internal and external torque, AP force, and flexion and extension angles are adopted from ISO 14243-1 [29]. The ISO is about test loading and displacement parameters for wear-testing machines for wear of total knee-joint prostheses. The IE rotation restraint was 0.6 Nm per degree according to ISO 14243-1. A 1.5 times vertical load was applied on the CM2 to simulate overweight.

The nonlinear material property of polyethylene is used as the same as in the paper of Taylor and Barrett [30]. As reported in the papers [31, 32], that polyethylene insert thickness is a very important factor in the design of total knee replacement, the thickness affects the wear of polyethylene; therefore, three thickness of tibial polyethylene insert 6.8 mm, 9.6 mm, and 12.3 mm were also compared for normal and overweight gait loads in this paper. The material properties in the two models are listed in Table 1.

## 3. Results and Discussion

Compared with Halloran's research work [27], the maximum posterior translation of tibial components obtained in this paper was 5.89 mm that is higher than the 5 mm in Halloran's research. The maximum internal rotation of the tibial tray obtained in this paper was 3.9° that is lower than the 4.6° in Halloran's research. The difference may be due to different implant geometries that were used; however, the results are comparable.

### 3.1. Influence of Body Weight on Stress Distribution in Distal Femur

**Figure 4:**
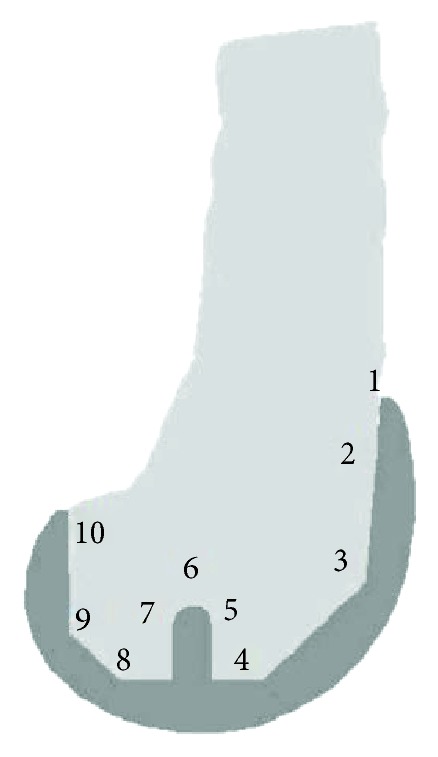
Distal femoral zones defined adjacent to the prosthesis.

Using the computer model CM1 shown in Figure 1, which simulated the hip and quadriceps forces, the stress distributions in the distal femoral bone were simulated for normal weight and 1.5 and 2 times normal weight, respectively. To show the effect of overweight clearly on the stresses in the distal femur during a gait cycle, stresses in different zones were compared; these zones are labelled and shown in Figure 4.

**Figure 5:**
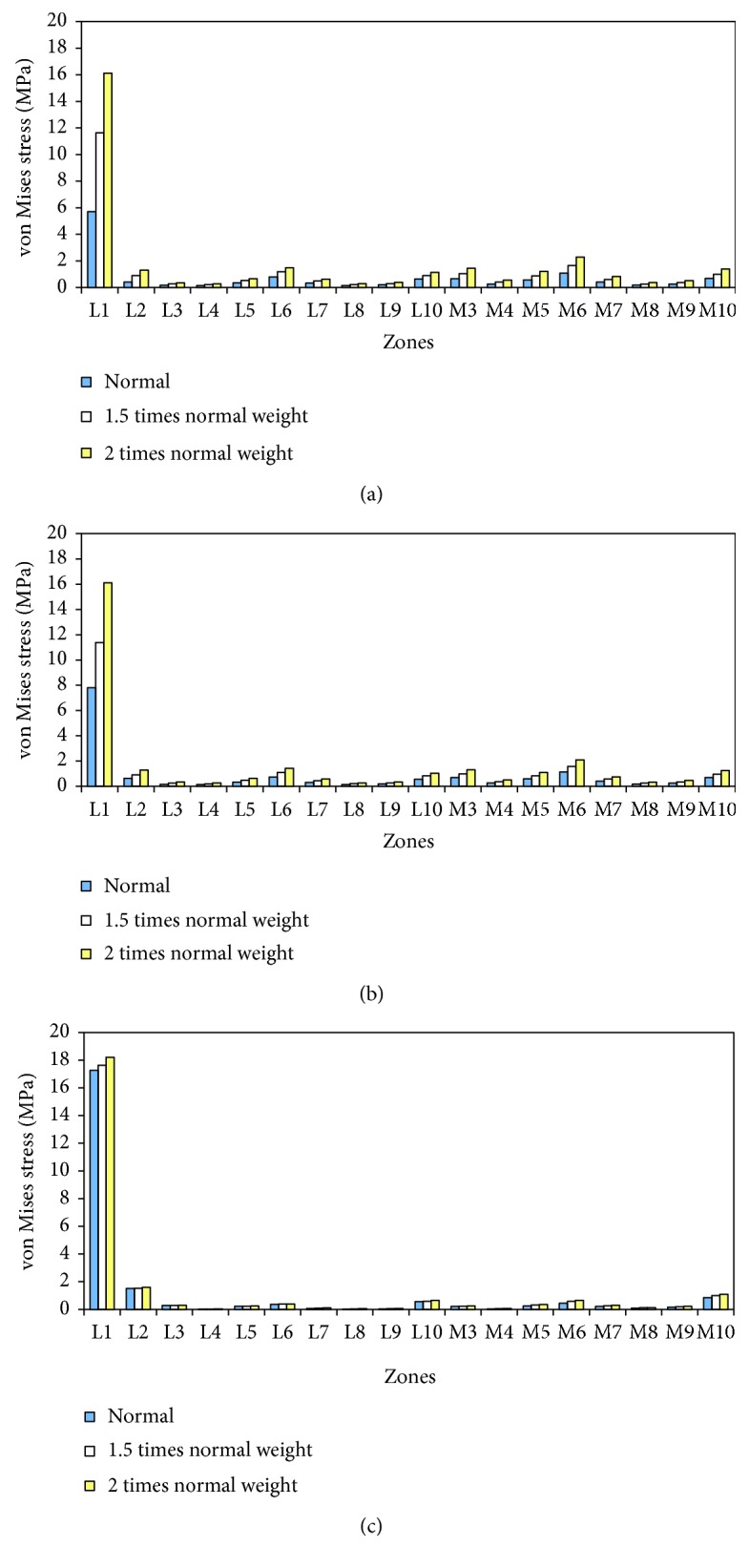
Comparison of stress distribution in distal femur after TKR with different body weight at (a) 15% of gait cycle, (b) 50% of gait cycle, and (c) 70% of gait cycle (L is for lateral condyle and M for medial condyle).

The von Mises stress at different zones for normal weight and 1.5 and 2 times the normal weight is plotted and shown in Figure 5. The increase of stress levels in all zones in conditions of overweight can be seen in Figure 5. Stresses at 15% and 50% of the gait cycle increased approximately in proportion to weight because the vertical force was the major force component during the stance phase period. However, the increase of stresses in the bone at 70% of the gait cycle was not proportional to the body weight. It can therefore be concluded that the increase of stress for overweight patients is directly related to the vertical load on the knee joint during walking. It was noticed that the stress values in zone 1 is much higher than other zones in Figure 5; this is because the zone 1 is cortical bone and other zones are cancellous bone. The increase rate of stresses in zone 1 at 15% of gait cycle is higher than the increase rate of load. In this computer simulation of TKA during a walking gait cycle, the increase of stress in conditions of overweight may result in the failure of bone-implant interface.

The increased weight leads to higher forces at the interface between bone and TKA components, which increases the chance of component aseptic loosening. Bagsby et al. [33] studied TKA in morbidly obese patients; after comparing the outcomes of cemented and cementless TKA, they concluded that cementless TKA may be a good option for morbidly obese patients, because a long-term biologic interface may better tolerate the excess loads generated at the bone-metal interface. The analysis in this paper did show the increased stress values in bones next to the TKA components and agrees with the findings by Bagsby et al. [33].

### 3.2. Effect of Body Weight on Tibio-Femoral Contact Pressure and Tibial Polyethylene Thickness in Mitigating the Effect

**Figure 6:**
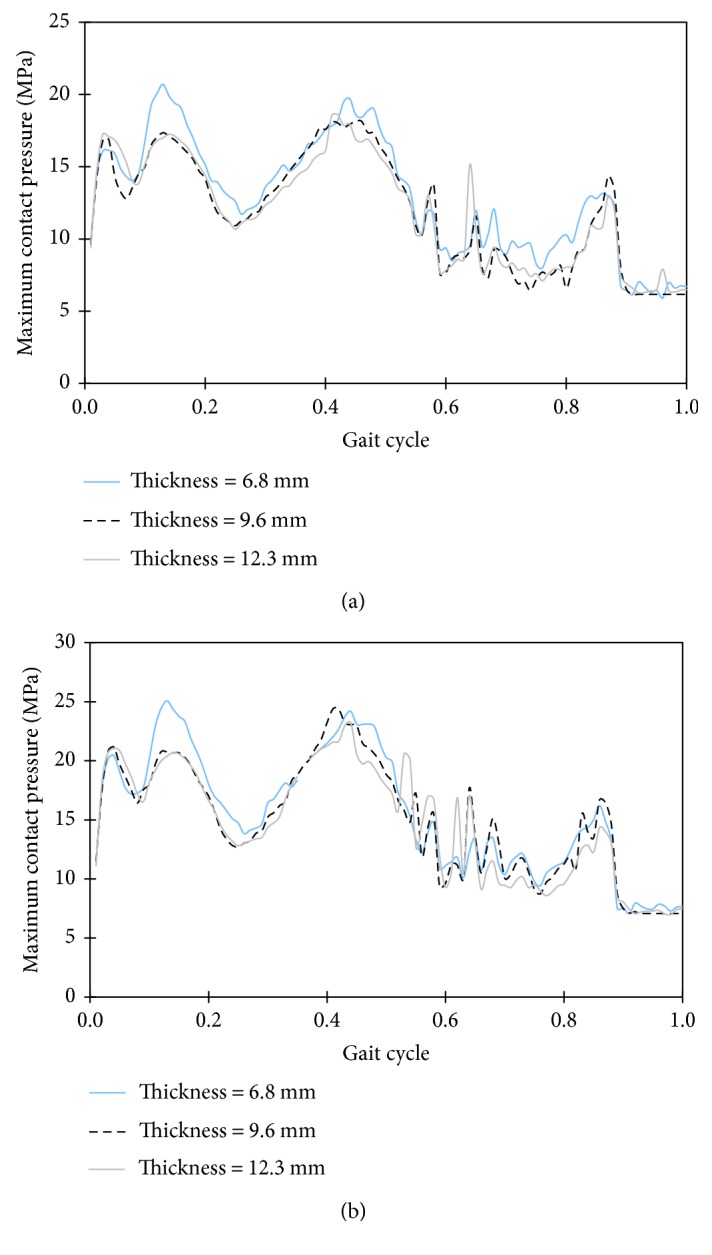
Comparison of maximum contact pressures on tibial polyethylene inserts under the load of (a) normal weight and (b) 1.5 times the normal weight.

The second computer model CM2 shown in Figure 3 simulates the TKR wear test environments as specified in the ISO standard. Therefore, this model is used to show the effect of overweight on the contact pressures in TKR. To investigate the influence of body weight on contact pressure in knee components, normal weight and overweight were simulated with the FE model CM2. An increased vertical load of 1.5 times the normal load was applied to simulate overweight. Taylor and Barrett [30] found that the thickness of tibial polyethylene insert affects the contact pressure on the polyethylene; the contact pressure decreased when the thickness of polyethylene increased. Therefore, three thickness of polyethylene were modelled to show how the thickness of the tibial insert could mitigate the effect of overweight.  Figure 6 shows the maximum contact pressure in the TKR with different thickness of polyethylene inserts under normal weight and overweight loads; contact pressure is shown as the function of a gait cycle. On the tibial polyethylene inserts, contact pressures in the 9.6 and 12.3 mm thickness design were lower than the 6.8 mm design under the normal weight and overweight loads. It can be seen that the maximum contact pressures are 25 MPa and 20.7 MPa on the 6.8 mm-thick tibial polyethylene insert, and these values were decreased to 20 MPa and 17 MPa on the 9.6 mm-thickness under 1.5 times and normal weight load, respectively. Therefore, the increased tibial polyethylene thickness could mitigate the adverse effect of increased weight in obese patients. The maximum contact pressure on the 12.3 mm thickness tibial polyethylene insert is nearly the same as in the 9.6 mm thickness; this means that an optimal thickness of tibial polyethylene insert could be determined for obese patients.

Liza et al. [34] studied the wear of a retrieval of a 10-year tibial polyethylene insert. As they mentioned, there were several damage modes to the polyethylene. Delamination and pitting which are related to fatigue wear were the most common feature of damage on tibial polyethylene inserts; the increased contact pressure will cause more wear. From the simulated results in this paper, it can be seen that contact pressures on the polyethylene insert were increased due to excess weight; however, the increased thickness of polyethylene decreased the contact pressure. When a thicker tibial polyethylene insert is designed in TKR, thinner tibial metal tray could be used to minimise bone cutting in the tibia. Recent research work on the effect of tibial metal tray thickness on tibial bone remodelling by Martin et al. [35] showed that there was a significant increase in the medial tibial bone loss in the metal tibial tray of 4 mm-thick than in the 2.7 mm-thick tibial tray. Therefore, thick tibial polyethylene inserts and thin metal tibial tray could be used in TKR for overweight and obese patients. Gait analysis [14] on obese individuals showed that they have shorter step length and larger ground reaction forces compared to lean individuals. The shorter step length requires more steps for obese patients to cover the same travel distance than normal people. The increased number of cycles and higher contact pressure are not good for the wear of polyethylene; therefore, more research on the TKR for obese patients should be carried out. Although this research were able to show the effect of overweight on TKR, it has limitations because the gait cycle of obese individuals was assumed to be similar to the normal people but with increased hip and quadriceps forces. To improve the finite element modelling of obese patients with TKR, gait measurement of this group should be conducted and patient-specific model should be created in future analyses.

The rehabilitation exercise after TKR is important in patient recovery and gain knee function. The computer models created in this research can be used to facilitate the simulation of the impact of obesity during postoperative exercise rehabilitation regimens following TKA, specifically it will identify if any particular exercise exacerbates stress/loading around the TKA components.

## 4. Conclusion

From the analysis of the total knee arthroplasty using the computer models, the stresses in the distal femoral bone were found to increase with body weight. Stresses at 15% and 50% of the gait cycle increased approximately in proportion to weight. The maximum stress in femoral bone was increased from 7.8 to 16 MPa when the body weight was doubled at 50% of the gait cycle. The higher stress indicates that the risk of femoral component migration will increase with the excess body weight. From the simulation of total knee components with increased load, the maximum contact pressures on the tibial polyethylene inserts were increased; this can lead to more wear in the tibial polyethylene inserts. Based on the simulation results in this paper and other researchers' findings, cementless total knee arthroplasty with thick tibial polyethylene insert and thin metal tibial tray may be a better option for overweight patients.

## Figures and Tables

**Table 1:** Material properties.

Material	Elastic modulus (MPa)	Poisson's ratio
Cortical bone	17,962	0.3
Metaphyseal cortical bone	7500	0.3
Cancellous bone 1	1091	0.3
Cancellous bone 2	400	0.3
Cancellous bone 3	100	0.3
Bone cement (PMMA)	2100	0.4
Cobalt-chrome alloy	193,000	0.29
Titanium alloy	110,000	0.33
